# Association of academic performance, general health with health-related quality of life in primary and high school students in China

**DOI:** 10.1186/s12955-020-01590-y

**Published:** 2020-10-12

**Authors:** Shengxiang Qi, Zhenzhen Qin, Na Wang, Lap Ah Tse, Huifen Qiao, Fei Xu

**Affiliations:** 1 Nanjing Municipal Center for Disease Control and Prevention, 2, Zizhulin, Nanjing, 210003 China; 2 School of Public Health and Primary Care, The Chinese University of Hong Kong, Hong Kong, China; 3 Nanjing Brain Hospital Affiliated to Nanjing Medical University, 264, Guangzhou Road, Nanjing, 210029 China; 4 Department of Epidemiology, School of Public Health, Nanjing Medical University, Nanjing, China

**Keywords:** Academic performance, General health, Health-related quality of life, Child Health Utility 9D, Students

## Abstract

**Purpose:**

To explore the association of academic performance and general health status with health-related quality of life (HRQoL) in school-aged children and adolescents in China.

**Methods:**

In this cross-sectional study conducted in 2018, students (grade 4–12) were randomly chosen from primary and high schools in Nanjing, China. HRQoL, the outcome measure, was recorded using the Child Health Utility 9D, while self-rated academic performance and general health were the independent variables. Mixed-effects regression models were applied to compute mean difference (MD) and 95% confidence interval (CI) of HRQoL utility score between students with different levels of academic performance and general health.

**Results:**

Totally, 4388 participants completed the study, with a response rate of 97.6%. The mean HRQoL utility score was 0.78 (SD: 0.17). After adjustment for socio-demographic attributes, physical activity, sedentary behavior, dietary patterns, body weight status and class-level clustering effects, students with fair (MD = 0.048, 95% CI 0.019, 0.078) and good (MD = 0.082, 95% CI 0.053, 0.112) self-rated academic performance reported higher HRQoL utility scores than those with poor academic performance, respectively. Meanwhile, students with fair (MD = 0.119, 95% CI 0.083, 0.154) and good (MD = 0.183, 95% CI 0.148, 0.218) self-assessed general health also recorded higher HRQoL utility scores than those with poor health, separately. Consistent findings were observed for participants by gender, school type and residential location.

**Conclusions:**

Both self-rated academic performance and general health status were positively associated with HRQoL among Chinese students, and such relationships were independent of lifestyle-related behaviors and body weight status.

## Background

Health related quality of life (HRQoL) is a subjective concept frequently applied to describe people’s physical, mental, social, psychological and functional aspects of health [1, 2]. HRQoL is not only widely used for clinical purposes but also employed in public health fields [3]. It is documented that children and adolescents under the age of 18 could self-perceive the HRQoL stably in the absence of significant health/life events [4], and lifestyle and behavior factors were associated with HRQoL among children and adolescents at population level [3, 5–10]. Recently, assessment of HRQoL status for children and adolescents who are at the critical stage of growth and development has become an important topic [11]. And HRQoL has also been used to assess the public health effects of population-based lifestyle and behavior intervention programs [12, 13].

While the key task for school-aged children and adolescents is academic/curriculum learning, some students also spend a great deal of time in leisure-time physical activity (PA) but many others possess sedentary behavior (SB) and poor habits of sleeping and dietary intake. These unhealthy lifestyle-related behaviors are risk factors for some chronic conditions (e.g., obesity and cardiovascular diseases) tracking from childhood to adulthood [14, 15]. HRQoL has been found to be associated with lifestyle-related behaviors and excess body weight [3, 5–10], but little is known about its link with the academic performance/achievement and general health status among children and adolescents.

One study from Argentina reported a positive association between academic performance and HRQoL among the 4th grade students (aged 9–12 years) [16]. In this Argentina study, participants were selected from primary schools in the city of Cordoba using random cluster sampling approach in 2014, with 533 students recruited and 494 included in the analysis [16]. The HRQoL was self-reported by students with KIDSCREEN-52, while academic performance was measured with participants’ final exam scores of two subjects (written language and math) [16]. Mixed-effects regression models were applied to investigate association between academic performance and HRQoL with adjustment for students’ age, gender, socioeconomic status and mother’s educational attainment, and school-level clustering effects [16].

With respect to the relationship between general health and HRQoL among children and adolescents, except for a report from psychology freshmen in a Netherlands university documenting a positive relationship between general health and HRQoL [17], the research among school-aged children is lacking. In this Netherlands study regarding general health and HRQoL, participants (N = 118) were volunteers of psychology freshmen (mean age ± standard deviation: 21.2 ± 5.4) from the university of Amsterdam, while HRQoL was self-reported using the instrument of RAND-36 (the Dutch version of SF-36) and general health was also self-rated by students with Likert response options [17]. Correlation between general health and HRQoL was examined using Spearman correlation coefficients [17].

China not only has the largest number of students in the world, but also holds a very highly competitive academic-learning environment for school children and adolescents. Due to the high expectation of a better academic performance from the schools and parents, Chinese students ought to spend much more time in curriculum learning but less time in recreational activities than their counterparts in Western societies [18, 19]. However, no studies have previously investigated the relationship between academic performance (AP), general health (GH) and HRQoL among general school students in China. Therefore, we conducted a population-based survey among primary and high school students in 2018 in Nanjing municipality of China, aiming to examine: (1) the relationship between academic performance, general health and HRQoL; and (2) whether or not such an association was independent of lifestyle-related behaviors and body weight status.

## Methods

### Study design and participants

This work was part of a population-based cross-sectional study “The Built Environment and Chronic Health Conditions: Children (BEACH-Children)”, which was conducted among school-aged children and adolescents from May to June of 2018 in Nanjing municipality of China [20]. The aims of our BEACH-Children study were to investigate: (1) association of built environment characteristics with obesity and obesity-related behaviors; (2) the relationship between lifestyle-related behaviors and HRQoL; and (3) the health literacy (HL) and its influencing factors among Chinese students.

With more than 8 million registered residents within 12 administrative districts [21], Nanjing had approximately 667,000 enrolled primary and high school students in the 2017–2018 academic year [22]. On average, there were about 40 students per class in Nanjing [22]. Students from the primary (grade 4–6 only), junior (grade 7–9) and senior high schools (grade 10–12) were the participants of our BEACH-Children study.

When estimating the sample size, we attempted to warrant sufficient statistical power (90%) to simultaneously examine the three associations in our BEACH-Children study. Considering the study design, sampling approach and statistical power, using parameters from previous studies [5, 23, 24], we estimated that: (1) 3902 participants would be sufficient for investigating association between obesity and built environment attributes; (2) 2588 for identifying association between HRQoL and lifestyle-related behaviors; and (3) 1560 for examining association between HL and influencing factors. Thus, an estimated sample size of approximate 3900 was adequate for achieving primary research objectives of our BEACH-Children study.

A multi-stage random cluster sampling method was applied to choose the participants in this study. Using random digits, we randomly selected one primary, junior and senior high school from all the corresponding schools from each of the 12 districts. We then randomly chose one class from each targeting grade (4–12) from the selected schools. Consequently, 108 classes from 36 schools were included in the study and all students (N = 4498) from these selected classes were the eligible participants. The flowchart of participants’ recruitment is shown in “Appendix 1”.

Written informed consent was obtained from both the schools and parents/guardians prior to the on-site survey. Academic and Ethics Committee of Nanjing Municipal Center for Disease Control and Prevention, China, reviewed and approved this study. All personal identifications were deleted before data analysis.

### Study variables

#### Outcome variable

The outcome variable was HRQoL, which was assessed using the validated Chinese version of Child Health Utility 9D (CHU9D-CHN) [10]. CHU9D was specifically developed to assess children/adolescents’ HRQoL for the purpose of health care treatment and public health intervention [25]. CHU9D-CHN was professionally translated from the original English version of CHU9D and cross validated for Chinese children/adolescents (age group: 9–18 years), showing good validity for measuring HRQoL among Chinese children/adolescents [10]. CHU9D comprises 9 domains, including worried, sad, pain, tired, annoyed schoolwork, sleep, daily routine, and ability to take part in activities [25]. The scoring algorithm of this instrument was also specifically developed for Chinese children and adolescents, which was reported in detail in our previous study [26]. In brief, the scoring algorithm for CHU9D was developed based on the best–worst scaling approach, and the CHU9D utility was recorded as “1” for the best state and “0” for the worst state. To investigate the association of academic performance and general health with HRQoL, we used HRQoL utility score as the continuous variable in the analysis.

### Explanatory variables

#### Academic performance

Academic performance was self-rated by students with a question “This question asks about your overall academic performance/achievement within your class. Just a general ranking, how do you think about your present academic performance compared to your classmates?”. There were five answer options: “very good”, “good”, “fair”, “poor” or “very poor”. The original 5-point likert options were further grouped into 3 levels: “good (very good or good)”, “fair” or “poor (very poor or poor)”. In China, each student is informed of the results (grade or score) of each exam, so the student has an idea of his/her general academic performance within the class. As the overall academic performance in an academic year may more realistically reflect student’s performance than the “objectively-observed” selected subject, we then justified to use self-rated overall academic performance in our study.

#### General health

General health was also self-assessed by students. Each participant was asked to self-assess the present status of his/her own general health with a question “Now, think about the present status of your general health, which one of the responses below would describe it most appropriate?” [5]. This self-assessed present general health question was embedded in the CHU9D-CHN as the item 10, which has been validated among Chinese children and adolescents in our previous study [5]. Originally, this question has five ordinal response options (“very good”, “good”, “fair”, “poor”, or “very poor”) [5, 27], which was combined into 3 categories for the analysis: good (very good or good), fair, poor (“very poor or poor”).

### Covariates

Students’ socio-demographic attributes, lifestyle-related behaviors and body weight status were considered as covariates. Socio-demographic attributes and lifestyle-related behaviors were self-reported by participants using standardized questionnaires. Lifestyle-related behaviors included physical activity, sedentary behavior, sleeping, and snacks and soft drinks consumption. Body weight and height were objectively measured by well-trained staff according to standard protocols.

#### Socio-demographic attributes

Socio-demographic attributes included participants’ age (continuous variable), sex (male or female), residential area (rural, suburban or urban), parental education (≤ 9 years, 10–12 years or 13+ schooling years) and school type (primary, junior high or senior high school).

#### Physical activity

Physical activity was measured with a validated instrument, the Item-specific Physical activity Scale for Chinese Children and Adolescents (I-PASCA) [28], based on participants’ self-recorded physical activity’s frequency and duration in the past 7 days. The intensity of physical activity was assessed using metabolic equivalent value (MET) according to the latest physical activity compendium: light-intensity (MET: < 3), moderate-intensity (MET: 3–6) or vigorous-intensity (MET: ≥ 6) PA [29].

Based on the weekly time of moderate-to-vigorous physical activity (MVPA) (moderate-PA time plus doubled vigorous-PA time), daily MVPA time was computed for each student. We defined sufficient PA as “at least 60 min/day MVPA plus ≥ 3 days/week muscle/bone-strengthening” according to the PA guidelines for Chinese children and adolescents [30]. In the present study, participants were classified as “not engaging in sufficient PA” or “engaging in sufficient PA”.

#### Sedentary behaviors

Screen and sleeping time were separately used to indicate sedentary behavior patterns. In the analysis, screen time was categorized into: “screen time < 2 h/day” or “screen time ≥ 2 h/day” [30], and total sleeping duration was grouped into “insufficient sleep duration” or “sufficient sleep duration” according to the recommendation for Chinese school students [31].

#### Dietary intake

Intake of snacks and soft drinks in the last 7 days was assessed using a validated food frequency questionnaire (FFQ) [32]. The weekly dietary consumption frequency was applied to classify participants into sub-groups for analysis. Based on the weekly median value of intake frequency for snacks (0.61 times/week) and soft drinks (1.43 times/week), snack and soft drink consumption were categorized as “lower level” or “upper level”, respectively.

#### Body weight status

For each participant, body weight was assessed to the nearest 0.1 kg and height to the nearest 0.01 m. Both body weight and height were measured twice and the mean value of the two readings was used to calculate body mass index (BMI). Therefore, body weight status were classified as “underweight/normal”, “overweight” or “obesity” according to the age- and gender-specific recommendations for Chinese children and adolescents [33].

### Data analysis

Chi-square tests were used to examine differences in percentages for selected participants’ characteristics between school types. Due to the non-normal distribution of HRQoL utility scores, two non-parametric tests, Mann–Whitney U and Kruskal–Wallis, were used to compare the differences of HRQoL utility scores (mean, standard deviation (SD)) between subgroups of interest. Residual analysis showed that class-level cluster effects existed in this study, we thus performed mixed-effects linear regression models to compute mean difference (MD) and 95% confidence interval (CI) of HQRoL between different levels of academic performance and general health, with adjustment of age, sex, residence, physical activity, sedentary behaviors, dietary intake, school type, parents’ educational level, and class-level clustering effects. Data were double-entered and cleaned using EpiData 3.1 (The EpiData Association 2008, Odense, Denmark) and analyzed with SPSS version 20.0 for Windows (SPSS Inc., Chicago, IL, USA). *P* < 0.05 was set as the level of significance for two tailed test.

## Results

Among all 4498 eligible participants, 4388 completed the questionnaire with a response rate of 97.6%. No difference was identified between the respondents and non-respondents in terms of age, sex and residence area. Participants recruited from the primary, junior high and senior high schools accounted for 36.5%, 33.2% and 30.3%, respectively. The mean (SD) age was 13.9 (2.5) years and 49.8% were girls. More participants resided in rural (46.3%) than the suburban (22.7%) and urban areas (31.0%). There were 16.7% overweight and 11.0% obese students (Table 1).

**Table 1:** Selected characteristics of participants in this study

	Overall (N = 4388)	School type			*p* value^a^
		Primary school (n = 1602)	Junior high school (n = 1455)	Senior high school (n = 1331)	
Age					
Mean (SD^b^)	13.9 (2.5)	11.2 (0.9)	14.1 (0.9)	16.9 (0.9)	
Gender (%)					
Boys	50.2	53.9	50.9	45.1	< 0.001
Girls	49.8	46.1	49.1	54.9	
Residence (%)					
Rural	46.3	44.5	46.5	48.1	0.192
Suburban	22.7	24.5	22.2	21.1	
Urban	31.0	31.0	31.3	30.8	
Self-rated academic performance (%)^c^					
Poor	20.6	16.5	25.1	20.7	
Fair	34.6	30.9	32.1	41.7	< 0.001
Good	44.8	52.6	42.8	37.6	
Self-assessed general health status^d^					
Poor	3.9	2.7	3.5	5.6	
Fair	35.7	25.7	36.0	47.3	< 0.001
Good	60.5	71.5	60.5	47.1	
Body weight status (%)					
Non-overweight	72.3	67.8	73.4	76.6	
Overweight	16.7	17.8	16.9	15.2	< 0.001
Obesity	11.0	14.4	9.7	8.2	
Physical activity (%)^e^					
Sufficient	20.0	23.0	19.0	17.5	< 0.001
Insufficient	80.0	77.0	81.0	82.5	
Screen time (%)					
< 2 h/day	96.3	96.8	95.3	96.7	0.060
≥ 2 h/day	3.7	3.2	4.7	3.3	
Parental educational attainment					
< 9 years	38.9	47.2	26.7	42.4	
10–13 years	32.2	27.0	37.9	32.3	< 0.001
≥ 13 years	28.8	25.8	35.4	25.2	

^a^Chi-square test

^b^SD: standard deviation

^c^Good- excellent or good; Fair-not good and not poor; Poor- poor or very poor

^d^Good- very good or good; Fair- not good or not bad; Poor-poor or very poor

^e^Sufficient physical activity refers to at least 60 min/day moderate-vigorous intensity physical activity plus ≥ 3 days/week muscle/bone-strengthening; while insufficient physical activity means less than 60 min/day moderate-vigorous intensity physical activity or having no ≥ 3 days/week muscle/bone-strengthening

Table 2 presents HRQoL utility scores by selected socio-demographic characteristics, academic performance and general health status. The mean value of HRQoL score was 0.78 (SD: 0.17) for overall participants. A significantly positive gradient in HRQoL mean score was observed with the increasing level of academic performance (poor: fair: good = 0.73: 0.77: 0.82, p_trend_ < 0.001) and general health status (poor: fair: good = 0.63: 0.74: 0.82, p_trend_ < 0.001), respectively. Moreover, a similar pattern was consistently shown for subgroups stratified by gender, school type, and residence location (Table 3).

**Table 2:** Scores of HRQoL based on CHU9D among participants by selected socio-demographic characteristics, self-rated academic performance and general health status in this study

	HRQoL utility scores			*p* value^a^
	n	Mean	SD	
Overall	4388	0.78	0.17	N/A
Gender (%)				
Boys	2204	0.79	0.18	0.017
Girls	2184	0.78	0.17	
School type				
Primary	1602	0.84	0.16	< 0.001
Junior high	1455	0.78	0.16	
Senior high	1331	0.73	0.18	
Residence (%)				
Rural	2030	0.79	0.17	0.411
Suburban	997	0.78	0.18	
Urban	1361	0.78	0.18	
Self-rated academic performance (%)^c^				
Poor	905	0.73	0.19	< 0.001
Fair	1517	0.77	0.17	
Good	1966	0.82	0.16	
Self-assessed general health status^d^				
Poor	169	0.63	0.21	< 0.001
Fair	1566	0.74	0.18	
Good	2653	0.82	0.16	

^a^*p* values for non-parametric tests between socio-demographic characteristics and behavior patterns

^b^Body weight status was categorized based on age- and gender-specific recommendations for Chinese adolescents

^c^Good- excellent or good; Fair-not good and not poor; Poor- poor or very poor

^d^Good- very good or good; Fair- not good or not bad; Poor- poor or very poor

**Table 3:** Distribution of HRQoL scores based on CHU9D in participants with different self-rated academic performance and general health status by gender, school type and residence in this study

	Gender						School type								
	Boys (N = 2204)			Girls (N = 2184)			Primary (N = 1602)			Junior high (N = 1455)			Senior high (N = 1331)		
	n of participants	Mean (SD) of HRQoL	*p* value*	n of participants	Mean (SD) of HRQoL	*p* value*	n of participants	Mean (SD) of HRQoL	*p* value*	n of participants	Mean (SD) of HRQoL	*p* value*	n of participants	Mean (SD) of HRQoL	*p* value*
Self-rated academic performance (%)^a^															
Poor	500	0.73 (0.20)	< 0.001	405	0.72 (0.17)	< 0.001	264	0.77 (0.18)	< 0.001	365	0.72 (0.18)	< 0.001	276	0.69 (0.20)	< 0.001
Fair	711	0.78 (0.17)		806	0.76 (0.17)		495	0.83 (0.15)		467	0.77 (0.15)		555	0.72 (0.18)	
Good	933	0.82 (0.16)		973	0.81 (0.16)		843	0.86 (0.15)		623	0.81 (0.15)		500	0.76 (0.17)	
Self-assessed general health status^b^															
Poor	89	0.61 (0.23)	< 0.001	80	0.64 (0.18)	< 0.001	44	0.69 (0.15)	< 0.001	51	0.60 (0.25)	< 0.001	74	0.60 (0.19)	< 0.001
Fair	730	0.75 (0.18)		836	0.73 (0.17)		412	0.79 (0.17)		524	0.75 (0.16)		630	0.70 (0.18)	
Good	1358	0.82 (0.16)		1268	0.82 (0.15)		1146	0.86 (0.15)		880	0.80 (0.15)		627	0.77 (0.16)	

	Residence								
	Rural (N = 2030)			Sub-urban (N = 997)			Urban (N = 1361)		
	n of participants	Mean (SD) of HRQoL	*p* value*	n of participants	Mean (SD) of HRQoL	*p* value*	n of participants	Mean (SD) of HRQoL	*p* value*
Self-rated academic performance (%)^a^									
Poor	481	0.73 (0.18)	< 0.001	178	0.71 (0.20)	< 0.001	246	0.72 (0.19)	< 0.001
Fair	690	0.78 (0.16)		349	0.77 (0.17)		478	0.76 (0.17)	
Good	859	0.82 (0.16)		470	0.81 (0.17)		637	0.82 (0.16)	
Self-assessed general health status^b^									
Poor	72	0.62 (0.20)	< 0.001	35	0.62 (0.23)	< 0.001	62	0.62 (0.20)	< 0.001
Fair	730	0.74 (0.17)		349	0.74 (0.18)		487	0.74 (0.18)	
Good	1228	0.82 (0.15)		613	0.81 (0.16)		812	0.82 (0.16)	

^a^Good- excellent or good; Fair-not good and not poor; Poor- poor or very poor

^b^Good- very good or good; Fair- not good or not bad; Poor- poor or very poor

^*^p values for non-parametric tests between socio-demographic characteristics and behavior patterns

Table 4 displays the linear association between academic performance, general health and HRQoL scores among students after adjustment for potential confounding factors. Relative to those with poor self-rated academic performance, students with fair (MD = 0.048, 95% CI 0.019–0.078) and good self-rated (MD = 0.082, 95% CI 0.053–0.112) academic performance reported significantly higher HRQoL utility scores. Meanwhile, participants with self-assessed fair and good general health recorded an average increase of 0.119 (95% CI 0.083–0.154) and 0.183 (95% CI 0.148–0.218) unit in HRQoL score compared to those with poor self-assessed health. Similar scenarios were also observed among subgroups of gender, school type and residential location.

**Table 4:** Mixed-effects linear regression estimates for associations between academic performance, general health and HRQoL utility scores in participants

	Overall (N = 4388)						Gender						School type		
	Model 1			Model 2			Boys (N = 2204)			Girls (N = 2184)			Primary (N = 1602)		
	Mean (SE)	95% CI	*P*	Mean (SE)	95% CI	*P*	Mean (SE)	95% CI	*P*	Mean (SE)	95% CI	*P*	Mean (SE)	95% CI	*P*
Self-rated academic performance^a^															
Fair	0.048 (0.015)	0.019, 0.078	0.001	0.045 (0.011)	0.024, 0.066	0.000	0.054(0.012)	0.029, 0.079	0.000	0.035 (0.013)	0.008, 0.061	0.010	0.052 (0.019)	0.013, 0.090	0.009
Good	0.082 (0.015)	0.053, 0.112	0.000	0.074 (0.010)	0.053, 0.095	0.000	0.080(0.012)	0.056, 0.104	0.000	0.066 (0.013)	0.039, 0.092	0.000	0.073(0.019)	0.036, 0.111	0.000
Self-assessed general health status^b^															
Fair	0.119 (0.018)	0.083, 0.154	0.000	0.111 (0.013)	0.086,0.136	0.000	0.134(0.018)	0.098, 0.170	0.000	0.085 (0.018)	0.050, 0.120	0.000	0.091(0.024)	0.044, 0.138	0.000
Good	0.183 (0.018)	0.148, 0.218	0.000	0.171 (0.013)	0.146, 0.196	0.000	0.193(0.018)	0.157, 0.228	0.000	0.147 (0.018)	0.112, 0.182	0.000	0.154(0.023)	0.108, 0.200	0.000

	School type						Residence								
	Junior high (N = 1455)			Senior high (N = 1331)			Rural (N = 2030)			Sub-urban (N = 997)			Urban (N = 1361)		
	Mean (SE)	95% CI	*P*	Mean (SE)	95% CI	*P*	Mean (SE)	95% CI	*P*	Mean (SE)	95% CI	*P*	Mean (SE)	95% CI	*P*
Self-rated academic performance^a^															
Fair	0.055(0.018)	0.018, 0.091	0.005	0.032 (0.020)	−0.010, 0.073	0.129	0.036 (0.016)	0.005, 0.067	0.025	0.057 (0.019)	0.019, 0.095	0.004	0.046 (0.020)	0.006, 0.087	0.027
Good	0.075(0.018)	0.039, 0.112	0.000	0.072(0.021)	0.030, 0.113	0.001	0.064 (0.015)	0.033, 0.094	0.000	0.076 (0.019)	0.039, 0.114	0.000	0.082 (0.019)	0.041, 0.122	0.000
Self-assessed general health status^b^															
Fair	0.143(0.022)	0.099, 0.187	0.000	0.103(0.021)	0.061, 0.144	0.000	0.113 (0.019)	0.075, 0.150	0.000	0.115 (0.028)	0.059, 0.170	0.000	0.099 (0.022)	0.056, 0.142	0.000
Good	0.191(0.022)	0.148, 0.234	0.000	0.173(0.021)	0.131, 0.215	0.000	0.178 (0.019)	0.141, 0.215	0.000	0.170 (0.028)	0.115, 0.225	0.000	0.158 (0.022)	0.115, 0.201	0.000

Mixed-effects linear regression analysis with adjustment for age, gender, school type, residence, body weight status, parental educational attainment, physical activity, screen time, sleep time, consumption of sugar-sweetened beverage and fast foods, and class-level clustering effects

^a^Poor as the reference group

^b^Poor as the reference group

## Discussion

In this population-based study, we attempted to examine the relationship between academic performance, general health and HRQoL among school-aged children and adolescents in China. We observed a positive association between academic performance or general health and HRQoL among Chinese school students. And, such significant associations were independent of main lifestyle-related behaviors and body weight status.

It is difficult for us to make direct comparison between our findings and those documented previously, because very few studies, like ours, were designed to investigate the relationship between academic performance, general health and HRQoL among general student population. In our study, the finding of a positive relationship between academic performance and HRQoL was consistent with that from a study among primary school students in Argentina [16]. HRQoL and academic performance were measured with different instruments between the Argentina study and ours, but all of them were self-reported by participants. Although sample sizes differed substantially (our study vs. Argentina study: 4388 vs. 494), participants were recruited following the same approach (the random cluster sampling method) and data were analyzed using the same statistical analysis method in the two studies. However, more covariates were considered in our study than those in the Argentina study.

A positive link between general health and HRQoL in our study was in line with that observed in the Netherlands university students [17]. The participants, sampling approach, sample size, instruments, and data analysis method of the Netherlands study differed from those of our study. In the Netherlands study, there were only 118 university psychology freshmen (mean age = 21.2) who voluntarily took part in the survey and the Dutch version of SF-36 was used to measure participants’ HRQoL. As for data analysis, only Spearman correlation coefficients were calculated to investigate the link between the general health and HRQoL in the Netherlands university freshmen study [17]. In our study, participants were randomly selected from primary and high schools (mean age = 13.9) with a large sample size of more than four thousands. Moreover, mixed-effects regression models were employed to examine the association of general health with HRQoL in our study.

Considering limited evidence from the literature, more studies are urgently encouraged to examine associations between academic performance, general health and HRQoL among general student populations from different societies. In the future, more well-designed studies regarding association of academic performance and general health status with HRQoL from different social, cultural and educational context are desired to provide further evidence for better understanding of potential influence of academic performance and general health on HRQoL among general students from around the world.

There were some mechanisms behind the relationship between academic performance, general health and HRQoL among students. It is consistently documented that academic performance was positively associated with life-satisfaction and happiness among children and adolescents, while poor life-satisfaction or low-level of happiness might consequently influence self-perceived HRQoL [34–38]. Therefore, this might partially explain the positive association between academic performance and HRQoL for children and adolescents. With respect to explanations for the association of general health and HRQoL, it was found that people with poor self-assessed general health were more likely to experience depression [39], which in turn might result in poor academic learning ability for the students [40, 41]. Thus, these might, in part, account for the positive relationship between general health and HRQoL in our study.

Some merits of this study should be mentioned. First, the instrument used to assess HRQoL in our study was CHU9D, which was the only tool specifically developed to measure HRQoL among children and adolescents. CHU9D was developed based on cost-effectiveness analysis for health-care treatment and population-based public health intervention programs [25]. Meanwhile, other instruments available for measuring HRQoL of children/adolescents were originally designed for adults and then adapted for children and adolescents [5]. Second, our study is the first one to examine the associations of academic performance and general health with HRQoL among primary and high school students in China. Third, our participants were the representative sample of overall primary and high school students within urban and rural areas in regional China. Fourth, participants’ response rate was as high as 97.6%. And, HRQoL instrument and its scoring algorithm were validated for students in China. Furthermore, findings were interesting in that each of academic performance and general health showed a positive gradient association with HRQoL among participants in this study.

However, major limitations of this study should also be addressed. First, academic performance and general health were self-reported by participants, potential bias regarding self-assessment may be a concern. Second, due to the nature of cross-sectional study, no causal relationship could be concluded between academic performance, general health and HQoL among participants.

In conclusion, this study among school-aged children and adolescents in China demonstrated that academic performance or general health status was positively associated with HRQoL, and these associations were independent of lifestyle-related behaviors and body weight status. A similar pattern was consistently observed among subgroups stratified by school type, gender or residence area. This study informs important public health implications for academic researchers and policy-makers that school-based tailored curriculum education and health promotion campaigns might improve HRQoL for students.

## Figures and Tables

**Fig. 1:**
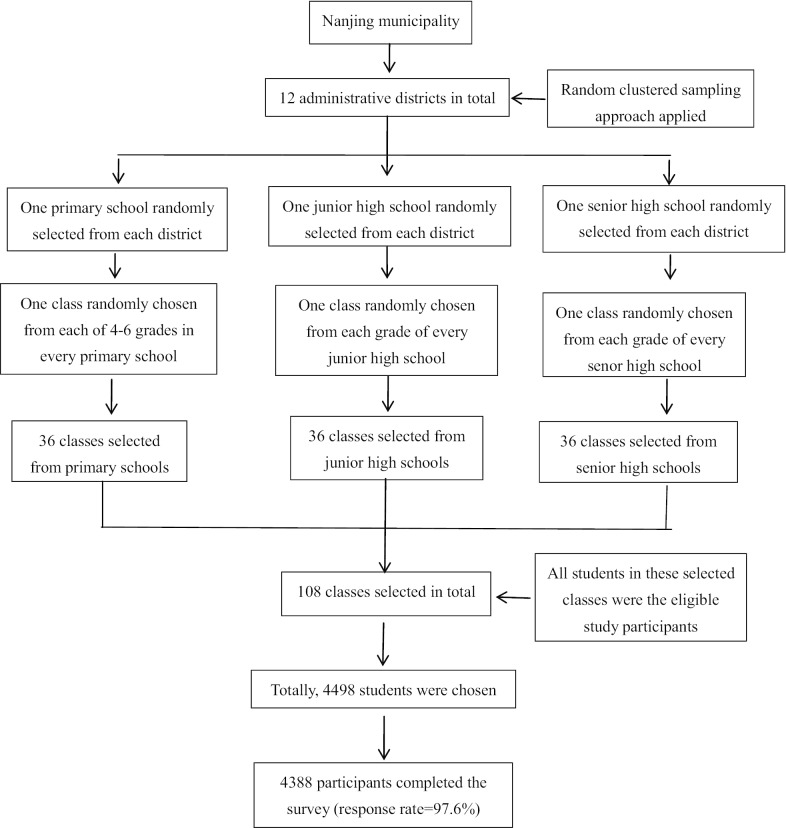
Flow chart for participants’ recruitment

## Data Availability

Data is available upon request to corresponding authors.

## Appendix 1

See Fig. 1.
